# Sparse conditional logistic regression for analyzing large-scale matched data from epidemiological studies: a simple algorithm

**DOI:** 10.1186/1471-2105-16-S6-S1

**Published:** 2015-04-17

**Authors:** Marta Avalos, Hélène Pouyes, Yves Grandvalet, Ludivine Orriols, Emmanuel Lagarde

**Affiliations:** 1 Univ. Bordeaux, ISPED, Centre INSERM U897-Epidémiologie-Biostatistique, F-33000 Bordeaux, France; 2 INSERM, ISPED, Centre INSERM U897-Epidémiologie-Biostatistique, F-33000 Bordeaux, France; 3 SISTM team, INRIA, F-33000 Bordeaux, France; 4 Univ. de Pau et des Pays de l'Adour, F-64012 Pau, France; 5 Univ. de Technologie de Compiègne, CNRS, Heudiasyc UMR7253, F-60203 Compiègne, France

**Keywords:** matched case-control study, case-crossover study, Lasso

## Abstract

This paper considers the problem of estimation and variable selection for large high-dimensional data (high number of predictors *p *and large sample size *N*, without excluding the possibility that *N < p*) resulting from an individually matched case-control study. We develop a simple algorithm for the adaptation of the Lasso and related methods to the conditional logistic regression model. Our proposal relies on the simplification of the calculations involved in the likelihood function. Then, the proposed algorithm iteratively solves reweighted Lasso problems using cyclical coordinate descent, computed along a regularization path. This method can handle large problems and deal with sparse features efficiently. We discuss benefits and drawbacks with respect to the existing available implementations. We also illustrate the interest and use of these techniques on a pharmacoepidemiological study of medication use and traffic safety.

## Background

Epidemiological case-control studies are used to identify factors that may contribute to a health event by comparing a group of cases, that is, people with the health event under investigation, with a group of controls who do not have the health event but who are believed to be similar in other respects. Logistic regression is the most important statistical method in epidemiology to analyze data arising from a case-control study. It allows to account for the potential confounders (factors independently associated with both the health outcome and the risk factors of main interest) and, if the logistic model is correct, to eliminate their effect.

Cases and controls are sometimes matched: every case is matched with a preset number of controls who share a similar exposure to these matching factors, to ensure that controls and cases are similar in variables that are related to the variable under study but are not of interest by themselves [1]. Matching is useful when the distributions of the confounders differs radically between the unmatched comparison groups. In these situations, the weight of confounding factors is so important that a simple adjustment does not guarantee a straightforward interpretation of results. The case-crossover design, in which each subject serves as his own control, is a particular matched case-control design [2,3]. The association between event onset and risk factors is estimated by comparing exposure during the period of time just prior to the event onset (case period) to the same subject's exposure during one or more control periods. This design inherently removes the confounding effects of time-invariant factors while it is still sensitive to the effects of time-varying risk factors [4,5]. The conditional logistic regression model is the standard tool for the analysis of matched case-control and case-crossover studies.

Big and/or high-dimensional data arise nowadays in many diverse fields of epidemiologic research such as registry-based epidemiology. An advantage of having a large sample size from registry data is the ability to study rare exposures, outcomes, or subgroups in a population large enough to provide sufficient precision [6]. However, the analysis of these studies has to be addressed using methods that appropriately account for their statistical and computational complexity. In what concerns matched case-control studies, matching by a high-dimensional propensity or stratification score are increasingly popular approaches to deal with high-dimensional confounding in epidemiological studies investigating effects of a treatment or exposure [7-10]. In these studies, a score is built from independent observations and then used to match data. Standard estimation methods accounting for data dependence due to matching, such as maximum-conditional likelihood are then applied and variable selection is performed by conventional selection procedures. Finding optimal subsets becomes an essential problem [11,12]. When high-dimensionality is related to the risk factors of main interest instead the potential confounders, regularization methods, such as the Lasso (*least absolute shrinkage and selection operator*) [13], have emerged as a convenient approach, but remain unfamiliar to most epidemiologists [14].

Our first implementation of the Lasso to conditional logistic regression was based on the correspondence between the conditional likelihood of conditional logistic regression and the partial likelihood of stratified, discrete-time Cox proportional hazards model (where cases are defined as events and controls are censored) [15,16].

This allowed the analysis of the pharmacoepidemiological case-crossover data of prescription drugs and driving described in Orriols and colleagues [17] for the older driver population. The same algorithm was independently proposed in two other high-dimensional matched case-control studies to identify association between

• Crohn's disease and genetic markers in family-based designs (such as case-sibling and case-parent) [18];

• specific brain regions of acute infarction and hospital acquired pneumonia in stroke patients [19].

Here, we describe a more efficient algorithm, directly targeting the optimization of the conditional likelihood. This algorithm is based on the IRLS (*iteratively reweighted least squares*) algorithm [20], which is widely used for estimating generalized linear models, and which can be applied with Lasso-type penalties. This line of approach was used in

• a matched case-control study to identify association between DNA methylation levels and hepatocellular carcinoma in tumor-adjacent non-tumor tissues [21];

• the case-crossover study of prescription drugs and driving for the whole population [22].

In these two studies, as well as in [23], the algorithms are based on the penalized IRLS, solved as a weighted Lasso problem *via *cyclical coordinate descent [24,25]. However, they differ in their actual implementations, a major benefit of our proposal relying in the simplification of the calculations involved in the likelihood function. The resulting gain in efficiency allows for the processing of large-scale datasets [22]. We detail these calculations here and illustrate their interest on the pharmacoepidemiological study that originally motivated these algorithmical developments.

## Methods

### Conditional likelihood

We are interested in the relationship between a binary outcome *Y *and several risk factors *U *= (*U*_1_, . . . , *U_p_*). We assume that subjects are grouped into *N *strata (corresponding to matched sets), consisting in one case (*Y_in _*= 1) and *M *controls (*Y_ln _*= 0, *l *≠ *i*) each one having a *U *value: For subject *i *of stratum *n*, the vector of observations is **u***_in _*= (*u_in_*1, . . . , *u_inp_*), *i *= 0, 1, . . . , *M , n *= 1, . . . , *N *.

Denote by *π_in _*= *π*(**u***_in_*) the probability of event for the *i*-th subject of the *n*-th stratum. We model the dependence of the probability of disease on the risk factors values via the logistic model, supposing that each stratum has its own baseline odds of disease, which may differ across strata:

(1) $$\text{logit}\left(\pi_{in}\right)=\alpha_{n}+u_{in}\beta ,$$

where *α_n _*are coefficients representing the global effect of matching factors on the response; and coefficients ***β ***= (*β*_1_, . . . , *β_p_*)*^t ^*express the log odds ratios corresponding to the risk factors. When the differences among strata are not relevant (in the sense that matching factors are potential confounders, but are not the potential risk factors of interest), we just need to estimate ***β***. Therefore, strata-specific parameters *α_n _*are eliminated from the likelihood by conditioning on the fact that exactly one subject in every matched case-control set is a case. Consider the *n*-th stratum, the unconditional probability of observing the occurrence of the event only in the *i*-th subject is:

(2) $$\pi_{in}\times \underset{l\neq i}{\prod }\left(1-\pi_{\text{ln}}\right)=\frac{\pi_{in}}{1-\pi_{in}}\overset{M}{\underset{l=0}{\prod }}\left(1-\pi_{ln}\right).$$

Under the logistic model, the conditional probability that within a matched set, the assignment of the *M *+ 1 values is given by:

(3) $$\frac{\frac{\pi_{in}}{1-\pi_{in}}\prod_{l=0}^{M}\left(1-\pi_{ln}\right)}{\sum_{l=0}^{M}\frac{\pi_{ln}}{1-\pi_{ln}}\prod_{i=0}^{M}\left(1-\pi_{in}\right)}=\frac{\frac{\pi_{in}}{1-\pi_{in}}}{\sum_{l=0}^{M}\frac{\pi_{ln}}{1-\pi_{ln}}}=\frac{e^{u_{in}\beta }}{\sum_{l=0}^{M}e^{u_{ln}\beta }}.$$

We use the convention that all cases are indexed by *i *= 0 and all controls are indexed by *i *∈ {1, . . . , *M*} (*Y*_0*n *_= 1 and *Y_in _*= 0, *i *≠ 0). When *M *= 1 (that is 1:1 matching), the likelihood evaluated at ***β ***simplifies to:

(4) $$L\left(\beta ,\;D\right)=\overset{N}{\underset{n=1}{\prod }}\frac{e^{u_{0n}\beta }}{e^{u_{0n}\beta }+e^{u_{1n}\beta }}=\overset{N}{\underset{n=1}{\prod }}\frac{1}{1+e^{-\left(u_{0n}-u_{1n}\right)\beta }}=\overset{N}{\underset{n=1}{\prod }}\frac{1}{1+e^{-x_{n}\beta }},$$

where **x***_n _*= **u**_0*n *_*− ***u**_1*n*_, *D *= {(**x***_n_*, 1)}_*n *= 1,...,*N *_. Thus, in a 1:1 matched case-control design, the conditional likelihood is identical to the unconditional likelihood of the binary logistic model with **x***_n _*as covariates, no intercept, and a constant response equal to 1. When *M >*1 (that is 1:M matching), it will be useful in the algorithms introduced below to rewrite the likelihood function:

(5) $$L\left(\beta ,\;D\right)=\overset{N}{\underset{n=1}{\prod }}\frac{e^{u_{0n}\beta }}{e^{u_{0n}\beta }+\sum_{l=1}^{M}e^{u_{ln}\beta }},$$

also in terms of differences:

(6) $$L\left(\beta ,\;D\right)=\overset{N}{\underset{n=1}{\prod }}\frac{1}{1+\sum_{l=1}^{M}e^{-\left({\text{u}}_{0n}-{\text{u}}_{ln}\right)\beta }}=\overset{N}{\underset{n=1}{\prod }}\frac{1}{1+\sum_{l=1}^{M}e^{-{\text{x}}_{ln}\beta }},$$

where **x***_in _*= **u**_0*n *_*− ***u***_in_, D *= {(**x***_in_*, 1)}_*n *= 1,...,*N *;*i *= 1,...,*M*_.

Usually, the parameters ***β ***are estimated by maximizing the conditional log-likelihood function log(*L*(***β**, D*)). However, maximum likelihood analysis may lead to an inflated variance and/or a biased estimation of log odds ratios in studies with a small number of strata or several unbalanced risk factors, especially when the number of covariates is large or the proportions are close to zero. Different methods have been developed, generally in low-dimensional settings, to correct bias while controlling for variance [26-31]. In moderate to high-dimensional settings, penalized methods such as the Lasso [13] have been proposed to reduce variance (to improve prediction accuracy) and to identify the subset of exposures that exhibit the strongest associations with the response [15,18,16]. The Lasso applied to conditional logistic regression consists in maximizing the conditional log-likelihood function penalized by the *L*^1 ^norm of the unknown coefficient vector, or equivalently, minimizing the negative objective function:

(7) $$\underset{\beta }{\text{min}}\left(-\text{log}\left(L\left(\beta ,D\right)\right)+\lambda ||\beta |{|}_{1}\right),$$

where *λ *is a regularization parameter, and $\left||\beta |{|}_{1}=\sum_{j=1}^{p}|\beta_{j}\right|$ is the *L*^1^-norm of coefficients. Then, the parameter estimator is:

(8) $${\hat{\beta }}_{D}^{\text{L}}\left(\lambda \right)=\underset{\beta }{\text{argmin}}\left(\overset{N}{\underset{n=1}{\sum }}\text{log}\left(1+\overset{M}{\underset{l=1}{\sum }}e^{-{\text{x}}_{ln}\beta }\right)+\lambda {\left||\beta |\right|}_{1}\right)$$

### Algorithms

The conditional likelihood in (6) derived for the conditional logistic model corresponds to the partial likelihood of the stratified, discrete-time Cox proportional hazards model. Standard survival data analysis software can be used for the analysis of a 1:M matched case-control study. Analogously, algorithms proposed for solving the Lasso for the Cox model allowing for stratification can be used.

For the particular design consisting in one case and one control, we may apply a penalized unconditional logistic regression. Indeed, as showed above, the conditional likelihood function simplifies dramatically resulting in the likelihood function for the unconditional logistic regression without intercept term applied to the differences of the predictors. The *L*^1 ^penalized logistic regression problem (7) is convex but not differentiable. This characteristic leads to greater difficulty in solving the optimization problem. There has been very active development on numerical algorithms. An extensive, although not exhaustive, review and comparison of existing methods can be found in [32,33].

The IRLS algorithm [20] uses a quadratic approximation for the average logistic loss function, which is consequently solved by a *L*^1 ^penalized least squares solver. This method is particularly easy to implement since it takes advantage of existing algorithms for the Lasso linear regression. We revisit here the IRLS algorithm proposed for solving the *L*^1 ^logistic model by [13,34,35]. These methods differ basically in the algorithm applied to resolve the Lasso linear step. The last authors applied the Lars-Lasso algorithm [36] to find a Newton direction at each step and then used a backtracking line search to minimize the objective value. They also provided convergence results. Essentially, we applied this proposal to the particular objective function arisen in 1:1 matching, but replacing the Lars algorithm by the cyclic coordinate descent algorithm [24,25]. We generalize then this approach to estimate the conditional logistic likelihood coefficients in 1:M matching (6). Sparsity-related works of other research areas have also explored the use and properties of IRLS [37-39].

#### IRLS-cyclic coordinate descent for the 1:1 matching

Denote by $f\left(\beta ,D,\lambda \right)=-\text{log}\left(L\left(\beta ,D\right)\right)+\lambda ||\beta |{|}_{1}=\overset{N}{\underset{n=1}{\sum }}\text{log}\left(1+e^{-{\text{x}}_{n}\beta }\right)+\lambda ||\beta |{|}_{1}$ the objective function in (7) and (8) with *M *= 1. In particular, the unpenalized objective function *f *(***β**, D*, 0) is noted *f *(***β***). Let **X **be the *N × p *matrix of the observed differences *x_nj _*= *u*_0*nj *_− *u*_1*nj *_, *n *= 1, . . . , *N , j *= 1, . . . , *p*; let **g**(***β***) and **H**(***β***) be the gradient and Hessian of the unpenalized objective function:

(9) $$\begin{array}{c} g\left(\beta \right)=-X^{t}{\left(\frac{e^{-x_{1}\beta }}{1+e^{-x_{1}\beta }},\;\ldots ,\;\frac{e^{-x_{N}\beta }}{1+e^{-x_{N}\beta }}\right)}^{t},\\ H\left(\beta \right)=X^{t}W\left(\beta \right)X, \end{array}$$

where

(10) $$W\left(\beta \right)=\text{diag}\left(\frac{e^{-x_{1}\beta }}{{\left(1+e^{-x_{1}\beta }\right)}^{2}},\;\ldots ,\;\frac{e^{-x_{N}\beta }}{{\left(1+e^{-x_{N}\beta }\right)}^{2}}\right),$$

is the matrix of weights. The Newton method consists in finding a step direction by computing the optimum ***γ***^[*k*] ^of the quadratic approximation at ***β***^[*k*] ^(the current point in the k-th iteration) as:

(11) $$\gamma^{\left[k\right]}=\beta^{\left[k\right]}-H^{-1}\left(\beta^{\left[k\right]}\right)g\left(\beta^{\left[k\right]}\right)$$

The next iterate is then computed using the step direction by a line search over the step size parameter *t*:

(12) $$\beta^{\left[k+1\right]}=\left(1-t\right)\beta^{\left[k\right]}+t\gamma^{\left[k\right]},$$

**Algorithm 1 **IRLS-cyclic coordinate descent algorithm for the 1:1 matching

1: Fix **X***_N × p_, λ ≥ *0, ***β***, 0 *≤ α*_1 _*≤ *0.5, 0 *< α*_2 _*<*1 and *τ >*0.

2: **while **the stopping criterion is not satisfied **do**

3:    Compute **W**^[*k*] ^and **z**^[*k*] ^using (10) and (13).

4:    Resolve (16) applying cyclic coordinate descent. Let ***γ***^[*k*] ^be the solution.

5:    Backtracking line-search:

       Initialize *t *= 1, set Δ***β***^[*k*] ^= ***γ***^[*k*] ^*− **β***^[*k*]^.

6:    **while **the stopping criterion is not satisfied **do**

7:       ***γ***^[*k*] ^= ***β***^[*k*] ^+ *t*Δ***β***^[*k*]^

8:       Check the stopping criterion: *f *(***γ***^[*k*]^) *≤ f *(***β***^[*k*]^) + *α*_1_*t ***g**(***β***^[*k*]^)*^t^*Δ***β***^[*k*]^

9:       **if **The stopping criterion not satified **then**

10:          *t *← *α*_2_*t*

11:       **end if**

12:    **end while**

13:    Compute ***β***^[*k*+1] ^= (1 *− t*)***β***^[*k*] ^+ *t**γ***^[*k*]^.

14:    Check the stopping criterion: $\frac{\left|f\left(\beta^{\left[k+1\right]}\right)-f\left(\beta^{\left[k\right]}\right)\right|}{\left|f\left(\beta^{\left[k+1\right]}\right)\right|}\leq \tau$

15: **end while**

where 0 *< t ≤ *1. Let **z**, the working response vector, be defined as:

(13) $${\left(z^{\left[k\right]}\right)}_{n}=x_{n}\beta^{\left[k\right]}+{\left(W^{\left[k\right]}\right)}_{n}^{-1}\frac{e^{-x_{n}\beta^{\left[k\right]}}}{1+e^{-x_{n}\beta^{\left[k\right]}}}=x_{n}\beta^{\left[k\right]}+\left(1+e^{-x_{n}\beta^{\left[k\right]}}\right).$$

Then the gradient, Hessian and the step direction in (11) can be reformulated as follows:

(14) $$\begin{array}{c} g\left(\beta^{\left[k\right]}\right)=-X^{t}W^{\left[k\right]}\left(z^{\left[k\right]}-X\beta^{\left[k\right]}\right),\\ \text{H}\left(\beta^{\left[k\right]}\right)=X^{t}W^{\left[k\right]}X,\\ \gamma^{\left[k\right]}={\left(X^{t}W^{\left[k\right]}X\right)}^{-1}X^{t}W^{\left[k\right]}z^{\left[k\right]}. \end{array}$$

Thus ***γ***^[k] ^is the solution to the weighted least squares problem:

(15) $$\gamma^{\left[k\right]}=\underset{\gamma }{\text{argmin}}{\left||(W^{\left[k\right]}\right)}^{1/2}(z^{\left[k\right]}-X\gamma {)||}_{2}^{2}.$$

Applying the same development to the penalized problem, we obtain that ***γ***^[k] ^is the solution to the penalized weighted least squares problem:

$$\gamma^{\left[k\right]}=\underset{\gamma }{\text{argmin}}\left|\right|{\left(W^{\left[k\right]}\right)}^{1/2}\left(z^{\left[k\right]}-X\gamma \right){\left|\right|}_{2}^{2}+\lambda {\left||\gamma |\right|}_{1}.$$

#### Generalization to the 1:M matching

As previously, the IRLS algorithm is applied to resolve (7)-(8). It iterates the following steps until convergence: first, for the current ***β***, update the matrix of weights and the working response vector, then, compute the vector that minimizes penalized weighted least squares problem, using cyclic coordinate descent; finally, perform a line search to determine the step size to update ***β***. The objective function in (7) is now $f\left(\beta ,D,\lambda \right)=-\text{log}\left(L\left(\beta ,D\right)\right)+\lambda ||\beta |{|}_{1}=\overset{N}{\underset{n=1}{\sum }}\text{log}\left(1+\overset{M}{\underset{l=1}{\sum }}e^{-{\text{x}}_{ln}\beta }\right)+\lambda ||\beta |{|}_{1}$

**Algorithm 2 **IRLS-cyclic coordinate descent algorithm for the 1:M matching

1: Fix **X***_N M × p_, λ ≥ *0, ***β***^0^, 0 *≤ α*_1 _*≤ *0.5, 0 *< α*_2 _*<*1 and *τ >*0.

2: **while **the stopping criterion is not satisfied **do**

3:    Compute **W**^[*k*] ^and **z**^[*k*] ^using (18).

4:    Resolve (19) applying cyclic coordinate descent. Let ***γ***^[*k*] ^be the solution.

5:    Backtracking line-search:

      Initialize *t *= 1, set Δ***β***^[*k*] ^= ***γ***^[*k*] ^*− **β***^[*k*]^.

6:    **while **the stopping criterion is not satisfied **do**

7:       ***γ***^[*k*] ^= ***β***^[*k*] ^+ *t*Δ***β***^[*k*]^

8:       Check the stopping criterion: *f *(***γ***^[*k*]^) *≤ f *(***β***^[*k*]^) + *α*_1_*t ***g**(***β***^[*k*]^)*^t^*Δ***β***^[*k*]^

9:       **if **The stopping criterion not satified **then**

10:          *t ← α*_2_*t*

11:       **end if**

12:       **end while**

13:       Compute ***β***^[*k*+1] ^= (1 *− t*)***β***^[*k*] ^+ *t**γ***^[*k*]^.

14:       Check the stopping criterion: $\frac{\left|f\left(\beta^{\left[k+1\right]}\right)-f\left(\beta^{\left[k\right]}\right)\right|}{\left|f\left(\beta^{\left[k+1\right]}\right)\right|}\leq \tau$

15: **end while**

Let **X **be the matrix of the observed differences *x_inj _*= *u*_0*nj *_*− u_inj _, i *= 1, . . . , *M , n *= 1, . . . , *N , j *= 1, . . . , *p*, now with *M N *rows and *p *columns. The gradient **g**(***β***) and Hessian **H**(***β***) of the unpenalized objective function have now the form:

(17) $$\begin{array}{c} g\left(\beta \right)=-X^{t}{\left(\frac{e^{-x_{11}\beta }}{1+\sum_{l=1}^{M}e^{-x_{l1}\beta }},\ldots ,\frac{e^{-x_{M1}\beta }}{1+\sum_{l=1}^{M}e^{-x_{l1}\beta }},\ldots ,\frac{e^{-x_{1N}\beta }}{1+\sum_{l=1}^{M}e^{-x_{lN}\beta }},\ldots ,\frac{e^{-{\text{x}}_{MN}\beta }}{1+\sum_{l=1}^{M}e^{-x_{lN}\beta }}\right)}^{t},\\ H\left(\beta \right)=X^{t}W\left(\beta \right)X, \end{array}$$

with the matrix of weights and the working response vector written now as:

(18) $$\begin{array}{c} W\left(\beta \right)=\text{diag}\left(\frac{e^{-x_{11}\beta }}{{\left(1+\sum_{l=1}^{M}e^{-x_{l1}\beta }\right)}^{2}},\ldots ,\frac{e^{-{\text{x}}_{M1}\beta }}{{\left(1+\sum_{l=1}^{M}e^{-x_{l1}\beta }\right)}^{2}},\ldots ,\frac{e^{-{\text{x}}_{1N}\beta }}{{\left(1+\sum_{l=1}^{M}e^{-x_{lN}\beta }\right)}^{2}},\ldots ,\frac{e^{-{\text{x}}_{MN}\beta }}{{\left(1+\sum_{l=1}^{M}e^{-x_{lN}\beta }\right)}^{2}}\right),\\ {\left(z^{\left[k\right]}\right)}_{in}=x_{in}\beta^{\left[k\right]}+{\left(W^{\left[k\right]}\right)}_{in}^{-1}\frac{e^{-x_{in}\beta^{\left[k\right]}}}{1+\sum_{l=1}^{M}{e^{-x_{ln}\beta }}^{{}^{\left[k\right]}}}={\text{x}}_{in}\beta^{\left[k\right]}+\left(1+\overset{M}{\underset{l=1}{\sum }}e^{-x_{ln}\beta^{\left[k\right]}}\right). \end{array}$$

With this *N M × N M *matrix of weights and working response vector of length *N M*, ***γ***^[k] ^is the solution to the penalized weighted least squares problem:

(19) $$\gamma^{\left[k\right]}=\underset{\gamma }{\text{argmin}}||{\left(W^{\left[k\right]}\right)}^{1/2}\left(z^{\left[k\right]}-X\gamma \right)|{|}_{2}^{2}+\lambda ||\gamma |{|}_{1}.$$

Notice that when using the likelihood function in (6) as a function of **x***_in_, i *= 1, . . . , *M , n *= 1, . . . , *N *, the matrix of weights is diagonal while, when using it as a function of **u***_in_, i *= 0, 1, . . . , *M , n *= 1, . . . , *N *, **W**(***β***) is nondiagonal, which complicates the matrix inversion problem in terms of computation.

## The regularization path

For a given value of *λ*, a certain number of predictors with non-zero regression coefficients are obtained by minimizing the *L*^1^-penalized negative log-likelihood. In general, the smaller *λ*, the more the penalty is relaxed, and the more predictors are selected. Inversely, the higher *λ*, the more predictors are eliminated. The regularization path is the continuous trace of the Lasso estimates of the regression coefficients obtained when varying *λ *from 0 (the maximum-likelihood solution for the full conditional logistic model) to a certain threshold, which depends on data, beyond which no predictors are retained in the model. In general, the amount of penalization on the *L*^1^-norm of the coefficients is chosen by computing, first, the regularization path of the solution to (7), as the regularization parameter varies. Then, the value of *λ *is estimated from a grid of values using an appropriate criterion. Unlike *L*^1^-regularization paths for linear models, paths for logistic models are not piecewise linear, approximate regularization paths should then be considered [36,40-42]. To construct the grid of *λ*-values, *λ*_max _*> . . . > λ*_min_, firstly, we calculate *λ*_max _and *λ*_min_, the smallest *λ *for which all coefficients are zero and the smallest *λ *for which the algorithm converge without numerical problems, respectively.

It can be shown that, if $\lambda >\underset{j\in \left\{1,\ldots \;,p\right\}}{\text{max}}|\frac{\partial f}{\partial \beta_{j}}\left(0\right)|$ then the directional derivatives of the $\lambda {∥\beta ∥}_{1}$ term at ***β ***= **0 **dominate and so ***β ***= **0 **is the minimizer of *f *(***β**, D, λ*) [41,42]. The evaluation of the gradient function **g**(***β***) at ***β ***= **0 **leads to $\lambda_{\text{max}}=\underset{j\in \left\{1,\ldots ,p\right\}}{\text{max}},|\frac{1}{M+1}\sum_{n=1}^{n}\sum_{l=1}^{M}x_{lnj}|$. We fix $\lambda_{\text{min}}=\in \lambda_{\text{max}}$.

Next, we generate *T *values equally spaced (on the linear or log scale) decreasing from *λ*_max _to *λ*_min_. For *λ*_1_=*λ*_max_, the initial vector of coefficients is set to ***β***0 = **0**. For each *λ_t_*, 1 *< t ≤ T *, the initial vector of coefficients is set to $\beta^{\left[0\right]}=\hat{\beta }\left(\lambda_{t-1}\right)$, i.e. the coefficient vector at convergence for the precedent *λ *value.

After this discretization, the optimal regularization parameter can be chosen by a model selection criterion such as cross-validation or the Bayesian Information Criterion (BIC) [43,44].

### Publicly available implementation

Several algorithms have been proposed for solving the Lasso for the Cox model [45,42-50]. Among those proposing a publicly available code, only the method proposed by Goeman [49] allows for stratification (implementation publicly available through penalized R-package). However, as discussed in [16], this implementation is not applicable to large datasets.

Among the efficient algorithms solving the Lasso for the logistic model, those proposed by [42] (consisting in a generalization of the Lars-Lasso algorithm described in [36]), [49] (based on a combination of gradient descent and Newton's methods), and [24] (based on a quadratic approximation followed by a cyclic coordinate descent method) have a publicly available R implementation (glmpath, penalized and glmnet packages, respectively). The glmpath package [42] did not accommodate models without intercept. The penalized package [49] allows for several practical options. In particular, a no-intercept Lasso logistic regression model can be fitted using the differences as independent variables and a constant response. However, though the Newton method has fast convergence, forming and solving the underlying Newton systems require excessive amounts of memory for large-scale problems. This package is optimized for situations with many covariates, but does not handle a large amount of observations. Finally, the glmnet package can deal efficiently with very large (sparse) matrices and has been shown to be faster than competing methods [33,24]. However, the logistic function of this package fails to converge when a constant response is used in the logistic model without intercept. A summary is presented in Table 1.

**Table 1:** Main publicly available R packages that solves the Lasso and other sparse penalties for the Cox, logistic or conditional logistic models (surveyed October 1st, 2014).

Package	1:1 matching?	1:M matching?	Amenable to processing of with grouping penalties	with large *N*	K:M matching?
*Logistic Model*					
glmpath [42]	NO	NO	NO	NO	NO
penalized [49]	YES	NO	NO	NO	NO
glmnet [24]	NO	NO	NO	NO	NO

*Cox Model*					
glmpath [42]	NO	NO	NO	NO	NO
penalized [49]	YES	YES	NO	NO	NO
glmnet [50]	NO	NO	NO	NO	NO

*Conditional Logistic*					
pclogit [21]	YES	YES	YES	NO	NO
clogitL1 [23]	YES	YES	NO	NO	YES
clogitLasso [43,51]	YES	YES	NO	YES	NO

Parallel to our work, Sun et al. [21] and Reid et al. [23] proposed an IRLS-cyclic coordinate descent algorithm to resolve the Lasso for (un)conditional logistic regression. While all these works rely on IRLS-cyclic coordinate descent, the objectives and the strategies implemented to address these objectives are different. The algorithm developed in [21] was implemented into the pclogit R package which can be downloaded at http://www.columbia.edu/~sw2206/. This algorithm is based on the optimization of the original unsimplified likelihood function (5) with a penalty that encourages the grouping encoded by a given network graph. The algorithm developed in [23] was implemented into the clogitL1 R package which can be downloaded from CRAN. The main concern of the authors is the extension to matched sets consisting in more than one case (denote *K *the number of cases per stratum) and *M >*1 controls, with large *K *and large *M *. In this situation, the conditional likelihood function has a more complicated form, and the authors apply a recursive formula to compute the likelihood and its derivatives exactly. This scheme results in involving intensive computations that are not amenable to the processing of large datasets.

Our R package clogitLasso is available at our institution's Web page, in the "links and downloads" menu, http://www.isped.u-bordeaux.fr/biostat. Two strategies are implemented. The first one, discussed in [16], is dedicated to small to moderate sample sizes. It is based on the stratified discrete-time Cox proportional hazards model and depending on the penalized package [49]. The second one, discussed in the present paper, is amenable to the processing of large datasets (large *N *). It directly targets the conditional logistic regression problem, relying on the lassoshooting package [25] for the application of cyclic coordinate descent. The lassoshooting package is particularly well adapted for large-scale problems and provides a no-intercept option. For example, large sparse data matrices (resulting from rare exposures), can be stored in a sparse format as well as the diagonal matrix of weights and working response vector. The Lasso solver lassoshooting proceeds with **X***^t^***WX **and **X***^t^***Wz **(of dimension *p × p *and *p × *1, respectively) instead of **W**^1*/*2^**X **and **W**^1*/*2^**z **(of dimension *N M × p *and *N M × *1, respectively). Other practical options are available, for example penalized and unpenalized (always included in the model) variables can be specified. The methods, model selection criteria and capabilities of clogitLasso are detailed in [51,44].

## Results

Medicinal drugs have a potential effect on the skills needed for driving, a task that involves a wide range of cognitive, perceptual and psychomotor activities. Nevertheless, disentangling their impact on road traffic crashes is a complex issue from a pharmacoepidemiological point of view because, between others, the large variety of pharmaceutical classes. A major approach relies on the use of population-registries data, such as those conducted in UK [52,53], Norway [54], France [17] or Finland [55]. We report the use of the algorithms detailed in this paper for exploratory analysis of the large pharmacoepidemiological data of prescription drugs and driving described in Orriols and colleagues [17].

### Data sources and designs

Information on drug prescriptions and road traffic accidents was obtained from the following anonymized population-based registries: the national health care insurance database (which covers the whole French population and includes data on reimbursed prescription drugs), police reports, and the national police database of injurious road traffic crashes. Drivers involved in an injurious crash in France, between July 2005 and May 2008, were included in the study.

Traffic crash data including information about alcohol impairment and drivers' responsibility for the crash were collected. When the breath test is negative (concentration *<*0.5 g/L), the driver is recorded as not being under the influence of alcohol. Responsibility was determined by a standardized method that assigns a score to each driver on the basis of factors likely to reduce driver responsibility (such as road, vehicle and driving conditions, type of accident, difficulty of the task involved, and traffic rule obedience, including alcohol consumption).

We consider all dispensed and reimbursed medicines to the drivers in the study, in the 6 months before the crash, coded by the WHO ATC (World Health Organization Anatomical Therapeutic Chemical) classification fourth level system. For each drug, the exposure period started one day after dispensing and the length of the exposure period was estimated from median values reported within a survey on drug prescription in France. This leaded to about 400 candidate binary predictors (exposure, coded 1, and unexposure, coded 0, to each medicinal drug).

The objective is to identify the relevant associations between the exposure to medicinal drugs and the risk of being responsible for an injurious non-alcohol related road traffic crash. We considered two study designs, each one addressing a different epidemiological question.

#### Individually matched case-control study

The epidemiological question is:

"What is different about at-fault drivers, if they are highly comparable to not at-fault drivers on external factors that may influence a road crash such as weather or road conditions?"

The purpose of the analysis is to compare exposure to medicinal drugs probabilities on the day of crash between at-fault drivers (cases) and not at-fault drivers (controls). Thus, we matched each case to one control (1:1 matching) on the basis of the date, hour and location of the crash. We also adjusted for potential confounders: age, sex and long-term chronic diseases, that is, factors that have shown to be associated with the risk of accident and may confound the impact of medicinal drugs on responsibility. These factors were forced in the models (unpenalized). Age was coded by using a discrete qualitative variable with seven categories: 18-24, 25-44, 45-64, 65-70, 70-75, 75-80, *≥*80 and then using dummy variables. Long-term diseases were defined by an administrative status in the French health care insurance database allowing full reimbursement of health care expenses related to 30 chronic diseases. Chronic disease was coded by using a binary variable: presence or absence of any fully reimbursed chronic diseases.

**Figure 1:**
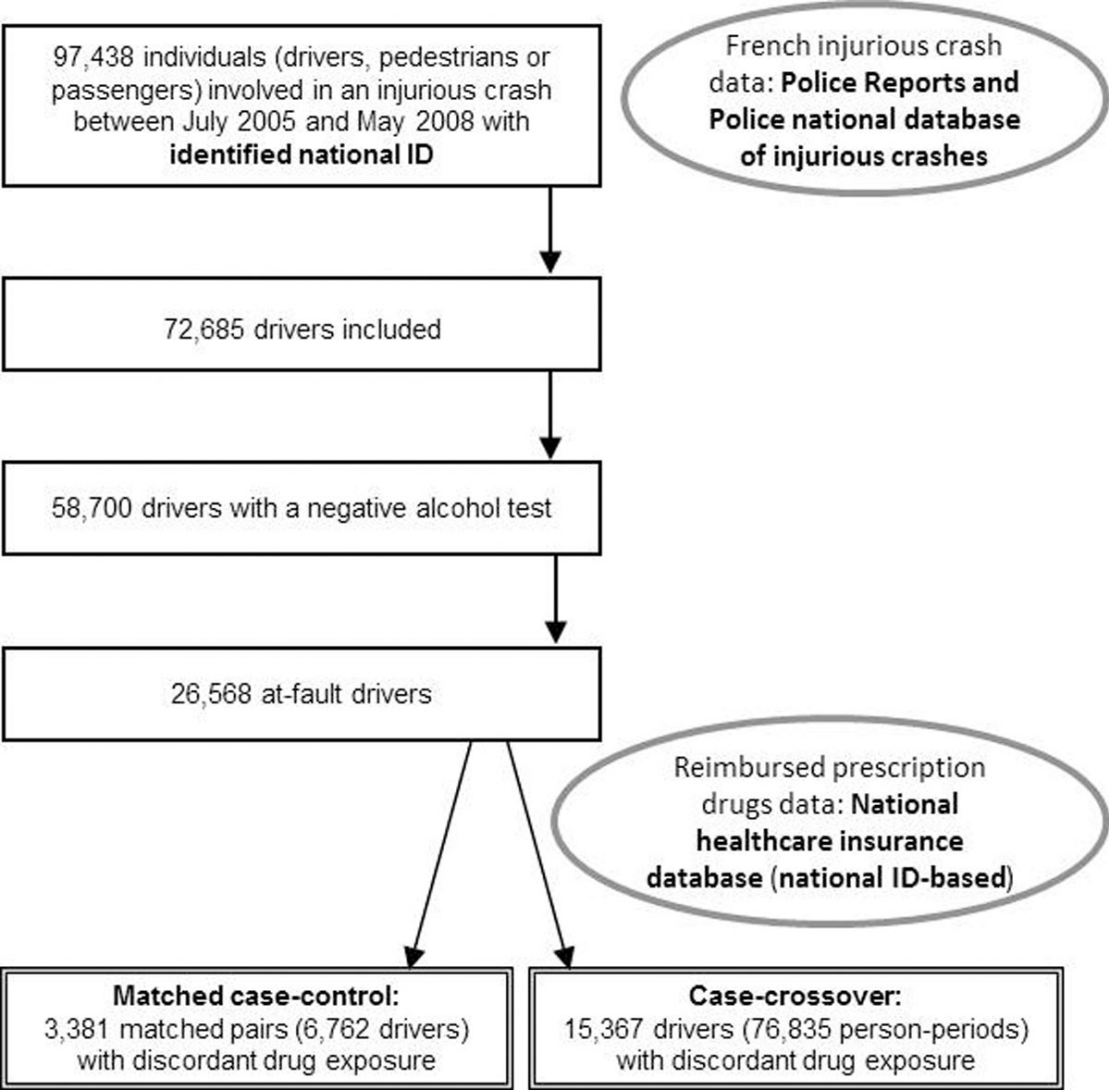
**Flowchart of the inclusion procedure**.

Of the 58,700 drivers with a negative alcohol test in the analytic database, 26,568 (45%) were considered responsible for their crash. After matching, 6,857 case-control pairs were highly comparable in terms of external factors, among them, 3,381 matched pairs showed different medicinal drug exposure (for at least one drug) on the day of the crash. Figure 1 shows the flowchart summarizing the selection of the subjects of the database. After eliminating medicines that have been little or not consumed (by less than 10 subjects), we get 189 binary predictors (in addition to the factors forced in the models).

#### Case-crossover study

The epidemiological question is:

"What is different about the day of the crash for at-fault drivers?"

The purpose of the analysis is to compare exposure to medicinal drugs probabilities on the day of crash (case period) and on the day one, two, three and four months prior to the crash date (control periods) for each at-fault driver. Thus, we matched each case period to four control periods (1:4 matching). Each period was separated from the next one by one month, the maximal duration of a treatment dispensed at the pharmacy in France, to avoid any residual effect of an exposure in one period on the following one.

Of the 26,568 at-fault drivers with a negative alcohol test in the analytic database, 15,367 (58%) showed different medicinal drug exposure (for at least one of the five periods; for at least one drug). Thus, 76,835 person-periods contributed to the estimation according to the flowchart in Figure 1, and they were described using the 189 binary predictors already mentioned above.

### Pharmacoepidemiological results

**Figure 2:**
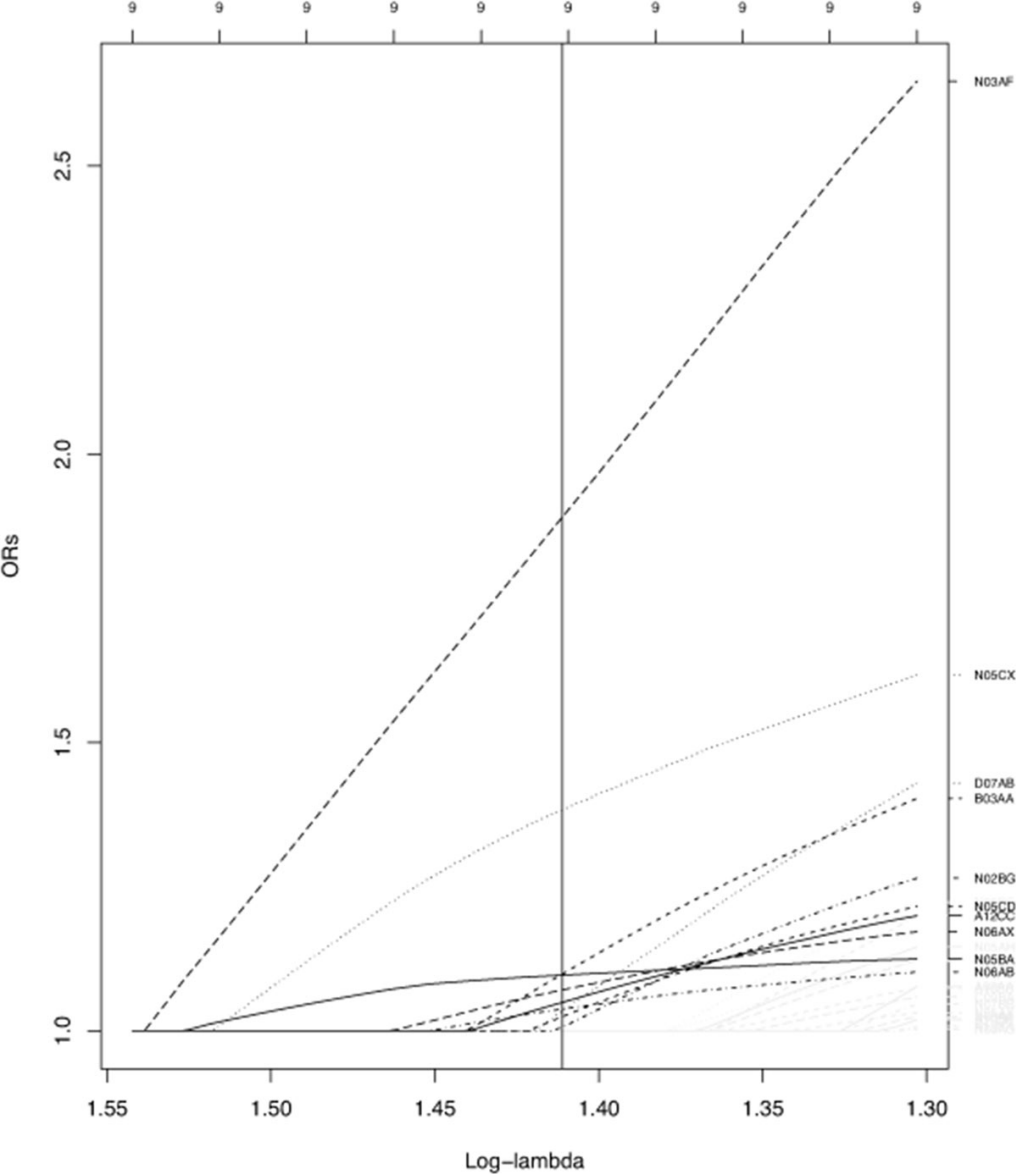
**Individually matched case-control study**. The Lasso regularization path as a function of *λ *for the paired case-control design. The black vertical line indicates the *λ *value optimizing the cross-validation criterion. Coefficient values of drugs selected are indicated in black, the others are in gray. Only drugs estimated to be risk factors (positive ${\hat{\beta }}_{j}$) are displayed. Potential confounders (sex, age, chronic disease), forced in the model, are omitted.

Although some drugs are usually prescribed together and correlation problems are possible, we observed only mild correlation, probably because the large sample size, then only the *L*^1 ^penalty was applied. We used 10-fold likelihood-based cross- validation to estimate the penalization parameter. Figure 2 shows the Lasso regularization path as a function of *λ *for the paired case-control study. Since all participants were involved in an accident, positive effects $\left({\hat{\beta }}_{j}>0\right)$ have not a direct interpretation as protective factors. Thus, they are not displayed.

Only four medicinal drugs were simultaneously selected as showing relevant associations with the risk of being at-fault for an injurious non-alcohol related traffic crash in both design studies (that is independently of the study design): Carboxamide derivative antiepileptics (N03AF), Benzodiazepine derivatives (N05CD), Other hypnotics and sedatives (N05CX), Antidepressants (N06AX). Odds ratio estimates are presented in table 2. The confounding underlying health conditions are not well controlled in our matched case-control study, however the case-crossover design inherently removes the confounding effects of time-invariant factors such as chronic health conditions. Thus, drug adversities may explain these results instead of chronic health-related complications. The effects of these four drugs on driving are well documented in the literature. In addition these medicines contain warning messages in relation to impairing driving ability. From a prevention perspective, it would be important to identify more precisely which populations are concerned by this at risk behavior. Further analyses should also be necessary to elucidate why these drugs appear to be related to an increased risk of at-fault crashes while other drugs from the same class do not. Such differences can simply be explained by a higher consumption, but other hypotheses are plausible.

**Table 2:** Odds ratio (OR) by study design.

ATC class second level	ATC class fourth level	Case-crossover	Matched case-control
Drugs for acid related disorders	A02BA	1.88	
	A02BX	1.19	
Drugs for functional gastrointestinal disorders	A03FA	1.24	
Laxatives	A06AD	1.37	
Mineral supplements	A12CC		1.10
	A12AX	1.57	
Antianemic preparations	B03AA		1.20
	B03BB	1.24	
Peripheral vasodilators	C04AX	1.15	
Antifungals for dermatological use	D01AE	1.13	
Corticosteroids	D07AB		1.16
Sex hormones and modulators of the genital system	G03CA	1.20	
Muscle relaxants	M03BX	1.23	
Analgesics	N02BG		1.09
Antiepileptics	N03AA	2.93	
	N03AF	1.34	2.11
	N03AX	1.19	
Psycholeptics	N05BA		1.11
	N05CD	1.37	1.09
	N05CX	1.01	1.46
Psychoanaleptics	N06AB		1.06
	N06AX	1.05	1.11
Drugs for obstructive airway diseases	R03BB	1.23	
Cough and cold preparations	R05DA	1.08	
Antihistamines for systemic use	R06AX	1.06	

Odds ratio estimates are displayed only for selected risk factors.

## Conclusion

We have developed a simple algorithm for the adaptation of the Lasso and related methods to the conditional logistic regression model. Our proposal relies on the simplification of the calculations involved in the likelihood function and the IRLS algorithm, that iteratively solves reweighted Lasso problems using cyclical coordinate descent, computed along a regularization path. As a result, this algorithm can handle large problems and deal with sparse features efficiently.

Problems related to high-dimensionality arise nowadays in many fields of epidemiological research (genetic, environmental or pharmacoepidemiology, for instance). In particular, we illustrate the interest of this methodology on the pharmacoepidemiological study of prescription drugs and driving that originally motivated these algorithmical developments.

The use of Lasso-related techniques is justified in this context as follows. First, regression models, with straightforward interpretation, are the most important statistical techniques used in analytical epidemiology. Thus, these techniques appear to be a good compromise between traditional and data-driven approaches since modeling is based on standard regression models, rather than a black-box. Second, controlling for potential confounding is a critical point in epidemiology, thus multivariate modeling approaches are preferable to separate univariate tests. Third, it is expected that only few drugs will be truly associated with the risk of being involved in a road traffic crash, thus sparsity-inducing penalties seem to be appropriate. It is also expected that most of these relevant drugs will have a weakly strength of association, however, only predictors with effect sizes above the noise level can be detected using Lasso-related techniques. Nevertheless, this limitation is shared by any model selection method [56-58].

## Competing interests

The authors declare that they have no competing interests.

## Authors' contributions

MA developed the algorithms and revised the R code, performed the analysis on the datasets and wrote the Manuscript. HP developed the R code. YG revised the algorithms, helped conduct the statistical/machine learning/bioinformatics literature review and revised the Manuscript. LO helped collect the data, performed the analysis on the datasets, interpreted the results of the analysis and conducted the epidemiological literature review. EL designed and supervised the epidemiological research, collected the data, and interpreted the results of the analysis. All authors read and approved the final manuscript.

## Declaration statement

Publication costs for this work were funded by the last author's institution: "Injury prevention and control" team of the French Institute of Health and Medical Research INSERM U897.

This article has been published as part of *BMC Bioinformatics *Volume 16 Supplement 6, 2015: Selected articles from the 10th International Meeting on Computational Intelligence Methods for Bioinformatics and Biostatistics. The full contents of the supplement are available online at http://www.biomedcentral.com/bmcbioinformatics/supplements/16/S6.
